# Mice Lacking the Alpha9 Subunit of the Nicotinic Acetylcholine Receptor Exhibit Deficits in Frequency Difference Limens and Sound Localization

**DOI:** 10.3389/fncel.2017.00167

**Published:** 2017-06-15

**Authors:** Amanda Clause, Amanda M. Lauer, Karl Kandler

**Affiliations:** ^1^Departments of Otolaryngology and Neurobiology, University of PittsburghPittsburgh, PA, United States; ^2^Center for the Neural Basis of Cognition, University of PittsburghPittsburgh, PA, United States; ^3^Center for Hearing and Balance, David M. Rubenstein Center, Department of Otolaryngology-Head and Neck Surgery, Johns Hopkins UniversityBaltimore, MD, United States; ^4^Department of Bioengineering, University of PittsburghPittsburgh, PA, United States

**Keywords:** auditory brainstem, lateral superior olive, development, acoustic startle, sound localization

## Abstract

Sound processing in the cochlea is modulated by cholinergic efferent axons arising from medial olivocochlear neurons in the brainstem. These axons contact outer hair cells in the mature cochlea and inner hair cells during development and activate nicotinic acetylcholine receptors composed of α9 and α10 subunits. The α9 subunit is necessary for mediating the effects of acetylcholine on hair cells as genetic deletion of the α9 subunit results in functional cholinergic de-efferentation of the cochlea. Cholinergic modulation of spontaneous cochlear activity before hearing onset is important for the maturation of central auditory circuits. In α9KO mice, the developmental refinement of inhibitory afferents to the lateral superior olive is disturbed, resulting in decreased tonotopic organization of this sound localization nucleus. In this study, we used behavioral tests to investigate whether the circuit anomalies in α9KO mice correlate with sound localization or sound frequency processing. Using a conditioned lick suppression task to measure sound localization, we found that three out of four α9KO mice showed impaired minimum audible angles. Using a prepulse inhibition of the acoustic startle response paradigm, we found that the ability of α9KO mice to detect sound frequency changes was impaired, whereas their ability to detect sound intensity changes was not. These results demonstrate that cholinergic, nicotinic α9 subunit mediated transmission in the developing cochlear plays an important role in the maturation of hearing.

## Introduction

The cochlea not only encodes sound and transmits auditory information to the brain but also receives abundant efferent innervation from the brain. Cholinergic neurons in the auditory brainstem give rise to the medial olivocochlear (MOC) system, which releases acetylcholine to activate nicotinic acetylcholine receptors on outer hair cells (OHCs; Elgoyhen et al., 1994, 2001; Guinan, 1996). The primary function of this cholinergic innervation is to adjust the gain of the cochlear amplifier (Guinan, 1986; Kim, 1986; Housley and Ashmore, 1991; Dallos et al., 1997), as it acts as an innate efferent feedback system that reduces auditory nerve activity, particularly in noisy environments (Winslow and Sachs, 1987; Guinan, 1996).

These MOC system-mediated effects are produced via suppression of cochlear responses to sound, which occurs through hyperpolarization of OHCs. Cholinergic hyperpolarization is accomplished by the activation of nicotinic acetylcholine receptors that consist of α9 and α10 subunits (Elgoyhen et al., 1994, 2001; Blanchet et al., 1996; Dulon and Lenoir, 1996; Evans, 1996; Fuchs, 1996; Lustig, 2006; Ballestero et al., 2011). Calcium influx through these receptors opens calcium-dependent potassium (SK2) channels, leading to potassium efflux and hyperpolarization of OHCs. OHC hyperpolarization then induces an electromotile response in these cells that decreases cochlear sensitivity to sound (Fuchs, 1996; Oliver et al., 2000; Goutman et al., 2005). α9 receptor subunits are crucial for functional acetylcholine receptors in hair cells because their genetic deletion eliminates cholinergic OHC hyperpolarization and MOC system-mediated suppression of cochlear activity (Vetter et al., 1999).

In the mature cochlea, MOC fibers exclusively synapse on OHCs. However, before the onset of hearing, MOC fibers transiently synapse on α9-containing cholinergic receptors on inner hair cells (IHCs), the primary sensory cells in the cochlea (Luo et al., 1998; Simmons and Morley, 1998; Bruce et al., 2000). As in OHCs, these transient cholinergic synapses are hyperpolarizing, and they inhibit spontaneous calcium action potentials that are generated in IHCS before the onset of hearing (Kros et al., 1998; Glowatzki and Fuchs, 2000; Goutman et al., 2005; Tritsch et al., 2010; Johnson et al., 2011; Roux et al., 2011). The effects of acetylcholine on immature IHC activity and its developmental roles are still debated (Wang and Bergles, 2015). However, in mice with homozygous deletion of the α9 nicotinic acetylcholine receptor subunit (α9KO mice), functional deafferentation of IHCs of their cholinergic inputs changes the temporal pattern, but not overall level, of spontaneous activity before hearing onset, as measured in neurons of the medial nucleus of the trapezoid body (MNTB), whose spiking closely follows the spontaneous activity patterns of auditory nerve fibers (Tritsch et al., 2010; Clause et al., 2014). Consistent with a role of cochlea-generated patterned activity before hearing onset in the developmental organization of central auditory circuits, α9KO mice show abnormal refinement of inhibitory MNTB projections to the lateral superior olive (LSO), resulting in degradation of the tonotopic precision of this pathway (Clause et al., 2014).

Although, α9KO mice lack a functional MOC system and exhibit central circuit abnormalities, these mice do not show generalized deficits in the detection and discrimination of auditory signals, as they have normal tone detection and intensity discrimination thresholds and exhibit normal hearing in noise (May et al., 2002). However, α9KO mice are more vulnerable to the development of temporal processing deficits, perhaps due to the abnormal structure and function of the endbulb of Held, a highly temporally precise auditory nerve synapse in the cochlear nucleus (Lauer and May, 2011; May et al., 2011). Interestingly, the impairment in temporal processing in α9KO mice is exacerbated by developmental exposure to louder environments, suggesting a role of MOC system-mediated cochlear gain adjustment in proper auditory development.

Acute MOC lesions in cats produce immediate sound localization deficits for stimuli presented in background noise in the vertical plane, but these deficits are ameliorated with continued practice on the task (May et al., 2004). MOC lesions in ferrets also impair learning of altered binaural cues in the horizontal plane (Irving et al., 2011). In humans, MOC feedback strength correlates with sound detection and discrimination ability in noise (Micheyl and Collet, 1996; Giraud et al., 1997; Micheyl et al., 1997; Kumar and Vanaja, 2004), including sound location discrimination (Andeol et al., 2011; Boothalingam et al., 2016). In addition, reduced MOC system function has been associated with auditory processing disorders in children (Muchnik et al., 2004; Sanches and Carvallo, 2006; Veuillet et al., 2007) as well as with word deafness (Kimiskidis et al., 2004).

Because α9KO mice show impaired circuit organization in the LSO, we investigated horizontal sound localization ability in a sound location discrimination task. In mice, this ability relies primarily on interaural level cues, which are first encoded in the LSO (Tollin, 2003; Lauer et al., 2011). In addition, because the decreased tonotopic precision of MNTB inputs to the LSO may reflect a general decrease of tonotopic map precision in α9KO mice, which could result in decreased sound frequency discrimination, we measured sound frequency difference limens in these mice using a prepulse inhibition (PPI) of the acoustic startle reflex paradigm (Clause et al., 2011). Our results indicate impaired sound localization ability and sound frequency difference limens in α9KO mice indicating a crucial role of cholinergic effect transmission in the cochlear in the maturation of hearing.

## Methods

### Subjects

The α9KO mice were originally described by Vetter et al. (1999) who donated them to The Jackson Laboratory (Bar Harbor, Maine), where they were bred onto a mixed CBA/CaJ and 129S6/SvEvTac background. Mice were obtained as live breeders, which were derived from JAX cryo-preservation. For the sound localization experiments, the female mice used were obtained from a breeding colony and housed in a quiet vivarium at Johns Hopkins University until behavioral testing, which lasted several months, was started at 6 weeks of age (Lauer et al., 2009). For the frequency and intensity difference limens experiments, α9KO mice and wild type control mice of the same genetic background, 129S6/SvEv, were obtained from a breeding colony at the University of Pittsburgh. In these experiments, both sexes were used for testing.

The experimental procedures for sound localization were approved by the Institutional Animal Care and Use Committee at Johns Hopkins University. The procedures for frequency difference limens were approved by the Institutional Animal Care and Use Committee at the University of Pittsburgh. All procedures were in accordance with the “Principles on Laboratory Animal Care” NIH guidelines.

### Sound localization

**Figure 1:**
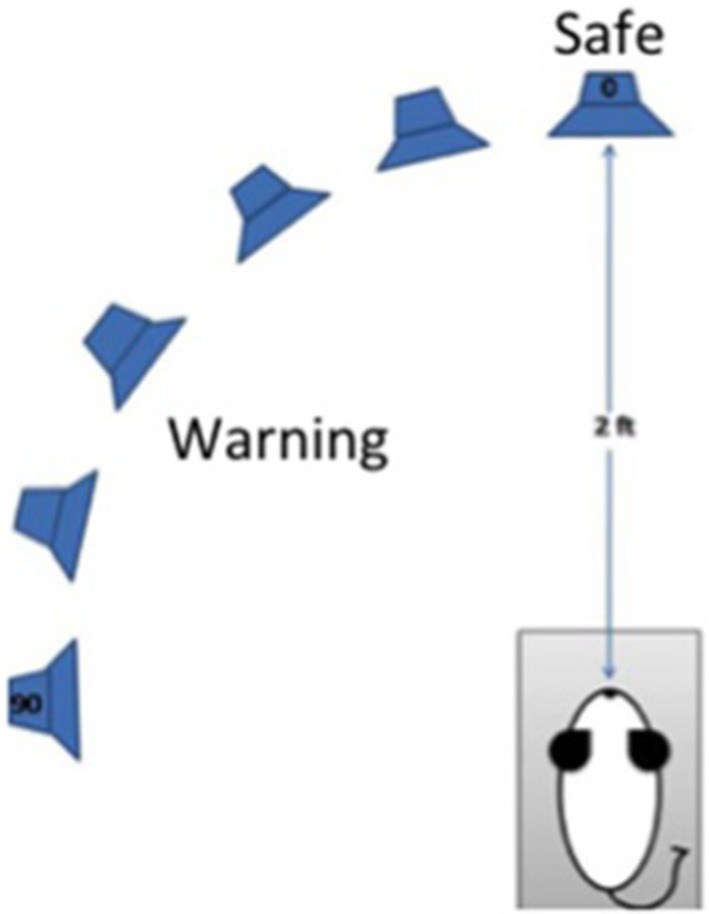
Schematic diagram of the procedures for testing discrimination of changes in the azimuthal location of a sound by mice. The mouse is placed in a wire cage trained to lick a spout to receive juice reward when “safe” sounds come from a speaker 0 degrees azimuth and elevation. The mouse must suppress licking when a sound is presented from any other location (20–90 degrees azimuth, 0 degrees elevation) to avoid a mild shock. Sounds are presented from an array of spectrally matched speakers 2 ft from the spout.

Procedures were identical to those reported by Lauer et al. (2011). A schematic of the approach is depicted in Figure 1. Briefly, mice were trained to discriminate sounds coming from directly in front (0 degrees azimuth and elevation) or from 20 to 90 degrees azimuth in the left hemisphere. An array of spectrally matched speakers (Vifa XT25TG30-04) was positioned 2 ft from the animal's head inside a double-walled sound-attenuating (Industrial Acoustics Company) which was booth lined with sound-absorbing anechoic foam (Pinta Acoustic) to reduce acoustic reflections. Speakers were located at 0, 20, 30, 50, 70, 90 degrees azimuth. The animal was tested in a small wire mesh cage to restrict movement. The cage was 5 cm wide × 11 cm long × 5 cm high. Additional wire mesh side guards were used to prevent the animal from turning to one side or the other. Water-restricted mice were trained to lick a spout to receive a juice reward when pairs of 240 ms broadband noise bursts separated by a 240 ms silent interval were presented at 0 degrees azimuth and elevation. Licking from the spout also served to keep the head in a calibrated position, which is essential for measuring minimum audible angles (MAAs). When the noise bursts were presented from another location in the horizontal plane, mice had to withdraw from licking the spout or receive a mild shock during the second burst in a stimulus pair. The amount of licking during 20 ms bins over a 400 ms period beginning 40 ms after the onset of the first burst was recorded for each stimulus condition. Bin counts were converted to hits and false alarms, and the criterion for suppression was adjusted for each subject to produce a false alarm rate of 16% which is typical for a well-trained subject in traditional operant procedures. Discrimination performance was based on d' values calculated based on the formula d' = z (hit rate) – z (false alarm rate). d' values were relatively insensitive to the 16% criterion for suppression because setting a subject's criterion higher would increase the number of hits, but also the number of false alarms. MAA was defined as the angular separation that produced a d' = 1.0.

### Frequency difference limens

A total of 92 mice were used for measuring frequency difference limens or intensity difference limens. Testing was either conducted just after hearing onset, at postnatal day (P) 14, or post-puberty, at P50. Mice tested at a young age were not tested again at a later time point, and mice tested for frequency difference limens were not also tested for intensity difference limens, or vice versa.

**Figure 2:**
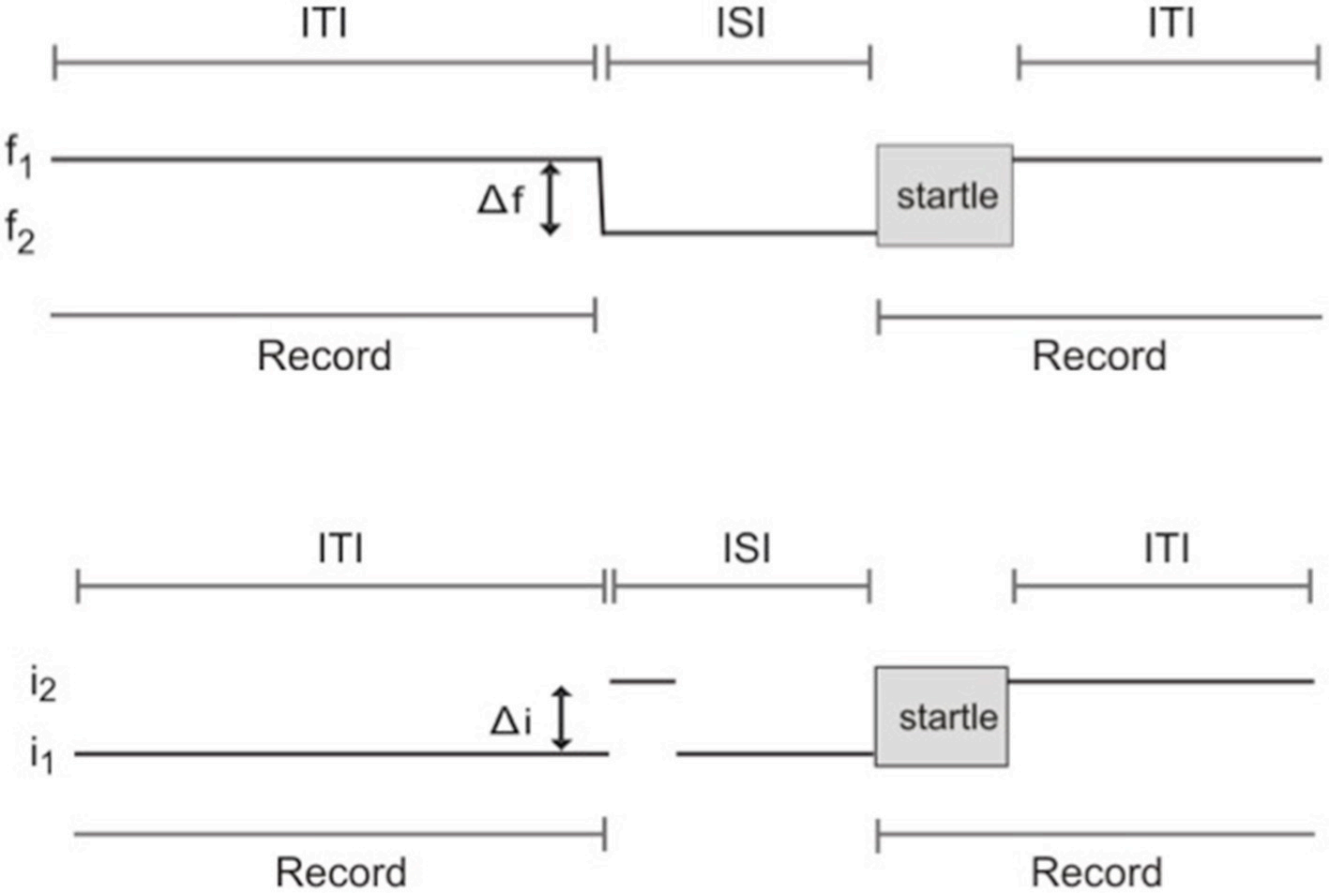
Schematic of trials used to test frequency change and intensity change limens using prepulse inhibition of the acoustic startle reflex. **(A)** Frequency change limens. f1, background frequency; f2, prepulse frequency; Δf, frequency change; ITI, inter-trial interval; ISI, inter-stimulus interval. **(B)** Intensity change limens. i1, background intensity; i2, prepulse intensity; Δi, intensity change; ITI, inter-trial interval; ISI, inter-stimulus interval.

Frequency difference limens testing was performed using an apparatus and methods as described previously (Clause et al., 2011). Briefly, mice were placed in the enclosure at the start of each session and allowed to acclimate to a constant background tone (f1: 16 kHz, 70 dB SPL) for 5 min. The acclimation period was then followed by “prepulse” and “startle only” trials. In prepulse trials, the prepulse stimulus comprised a change in frequency consisting of a 1 ms linear ramp from the background tone, f1, to the prepulse tone, f2, also at 70 dB SPL. The change from background, f1, to prepulse frequency, f2, took place in variable steps of size Δf, such that f2 = f1 − Δf. f2 was maintained for an 80 ms inter-stimulus interval (ISI). The ISI was then followed by the startle-eliciting stimulus (white noise at 120 dB SPL, duration 40 ms). At the conclusion of the startle stimulus, f1 was presented again until the prepulse stimulus of the next trial. In startle only trials, the prepulse stimulus consisted of a 1 ms ramp from f1 to f1, and thus maintained the command to change frequency, while not actually introducing a frequency change. All trials were separated by an 8–25 s inter-trial interval (ITI, length randomly selected) to prevent the animal from anticipating presentation of the startle stimulus. The trial schematic for frequency change detection testing is shown in Figure 2A.

Trials were divided into three blocks. Block one consisted of a series of startle-only trials to allow for short-term habituation to the startle stimulus. Block two contained prepulse trials randomly interleaved with an equal number of startle only trials. Prepulse trials were made up of seven classes, 10 trials each, where f2 = 15.92, 15.68, 15.47, 15.2, 14.4, 13.34, and 12.0 kHz. Block three consisted of a short series of startle-only trials to check for habituation over the course of the session.

During each trial, the vertical force exerted by the animal on the platform was measured during two 500 ms recording windows. The first recording window occurred immediately before the prepulse stimulus and provided a gauge of the animal's baseline activity. The second recording window began at the onset of the startle stimulus and measured the startle response of the animal to the white noise burst.

### Intensity difference limens

Intensity difference limen testing was performed using the same apparatus and a procedure similar to that used for frequency difference limens testing. The prepulse stimulus for intensity difference limens testing comprised a change in sound intensity. White noise at 70 dB SPL was used as the background (i1). The prepulse stimulus for prepulse trials comprised an intensity change of the background white noise, i1, to the prepulse intensity, i2, in variable steps of size Δi, such that i2 = i1+ Δi. Again, in startle only trials, the prepulse stimulus consisted of a transition from i1 to i1, maintaining the command to change intensity while not actually introducing an intensity change. The same acclimation period, startle stimulus, ISI, and randomized ITIs as those used for frequency difference limens testing were used for intensity difference limens testing. The same block design was also employed, with blocks one and three consisting of startle only trials, and block two containing the same proportion of prepulse trials randomly interleaved with an equal number of startle only trials. Prepulse trials were made up of five classes, 10 trials each, where i2 = 74, 78, 82, 86, and 90 dB SPL. A schematic of trials for intensity difference limens testing is shown in Figure 2B.

## Results

### Sound localization

**Figure 3:**
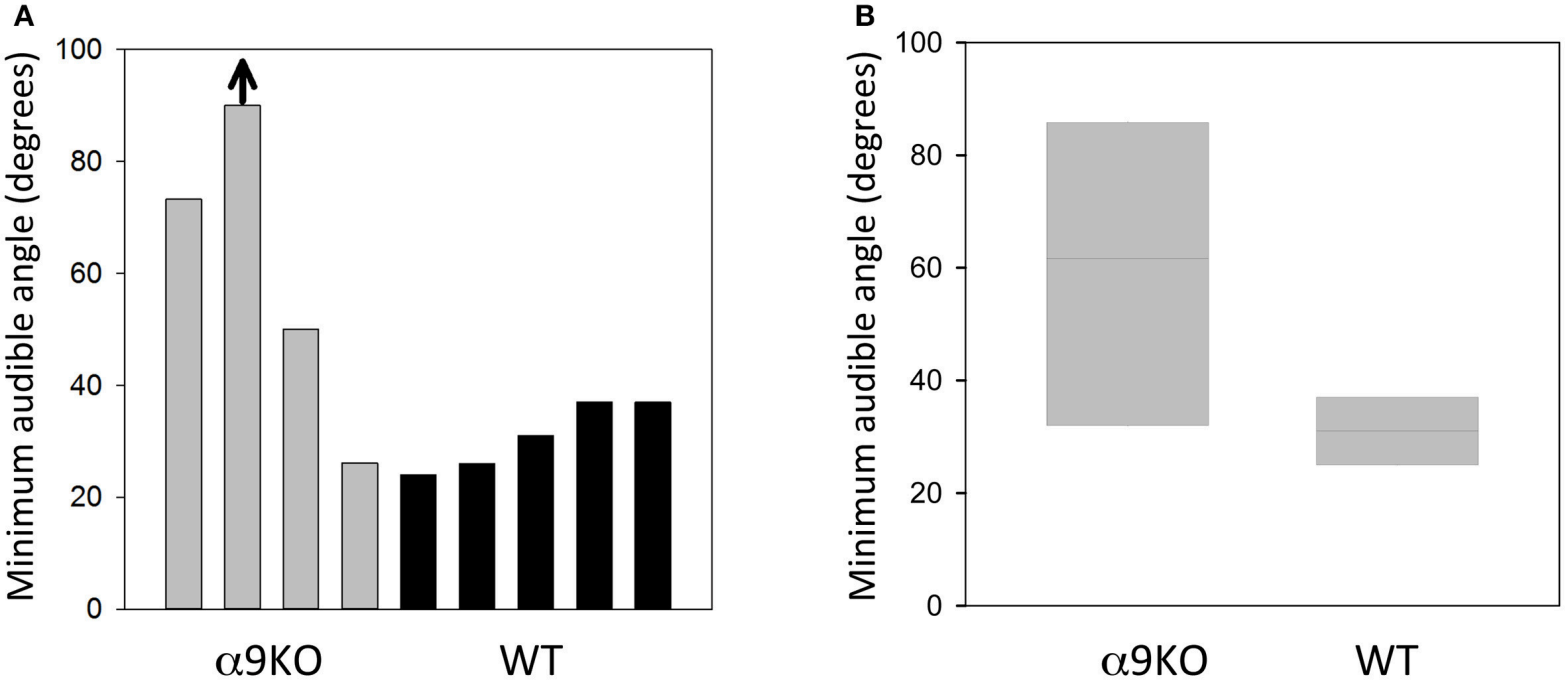
Sound localization performance in α9KO and WT mice. **(A)** Minimum audible angles from individual α9KO and WT mice are plotted to show the large variability in sound location discrimination performance. One animal performed below the threshold criterion of d' = 1.0 even for the largest angular separation tested (arrow). **(B)** Boxplots of minimum audible angles for α9KO and WT groups showing the median (line) and 25th (bottom box border) and 75th (top box border), percentiles.

The MAA for four α9KO mice and five wild type controls are shown in Figure 3. The average MAA for α9KOs was 60 degrees, whereas it was 31 degrees for wild types. The α9KO mice showed extremely variable performance. No threshold could be calculated for one α9KO as performance was below d' = 1.0 for all speaker locations, so a nominal MAA value of 90 degrees, the largest speaker separation tested, was assigned to this animal. This animal was able to learn the initial stages of the trained behavior, but could not perform the location discrimination above chance. In addition to the larger average MAA, the α9KO mice showed a much wider spread of scores than the wild type mice (26.2–90 and 24–37 degrees, respectively). Three out of four of the α9KO mice had larger MAAs than the wild types, but one α9KO mouse showed performance within the normal range. A Mann–Whitney *U*-test revealed that the difference between groups was not statistically significant (*U* = 3.5, *p* = 0.138) likely because of the single α9KO that showed a small MAA. For the largest angular separation, 90 degrees, d' values ranged from 0 to 2.2 in α9KO mice and 1.6–2.9 in WT mice, indicating greater performance variability in α9KO mice even for suprathreshold stimuli.

### Frequency difference limens

#### Frequency difference limens shortly after hearing onset

**Figure 4:**
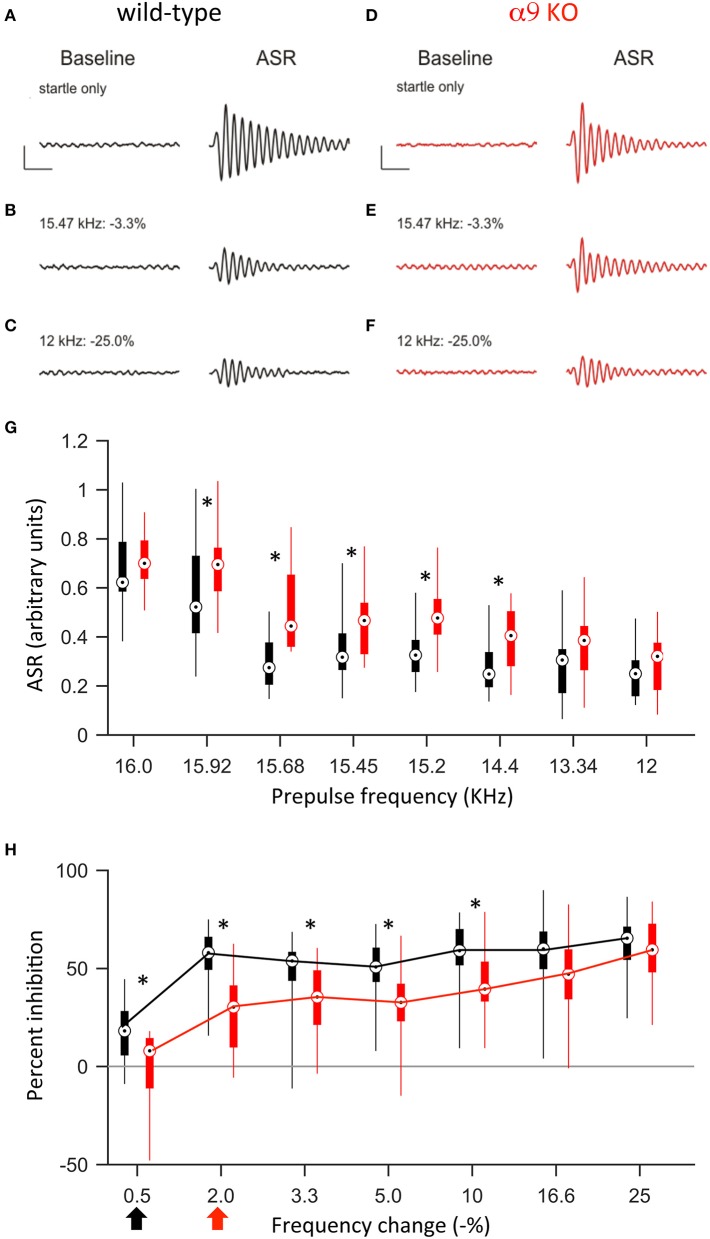
At P14, α9KO mice show impaired PPI for small, but not large, changes in frequency. **(A–F)** Examples of baseline activity and acoustic startle response for control (black; **A–C**) and α9 KO (red; **D–F**) mice. Traces represent the force measured on the platform during the first 500 ms recording period, “baseline,” and force measured during the second 500 ms recording period, “ASR” for **(A,B)** startle only trials and **(C–F)** prepulse trials with frequency steps of increasing size. Prepulse trials are labeled with the frequency of the prepulse and magnitude of the resulting frequency change. For each trial, the maximum force recorded during the second recording period, in either the positive or negative direction, was reported as the ASR. Scale bars, 0.4 arbitrary units of force, 100 ms. **(G)** ASR amplitudes for control (black) and α9 KO (red) mice for each trial type. Data are presented as boxplots with boxes representing the 25th and 75th percentiles and vertical lines indicating full data range. Asterisks indicate that ASR magnitude for α9 KO mice is significantly different than ASR magnitude for control mice for that class of trials [Two way ANOVA, *F*_(1, 248)_ = 36.5, *p* < 0.001, main effect of genotype, Holm–Sidak *post-hoc* pairwise comparison, *t* = 6.043, *p* < 0.001]. *n* = 20 control and 13 α9 KO animals. **(H)** Percent inhibition of the ASR caused by a prepulse frequency change of various magnitudes. Arrows indicate discrimination threshold, the smallest frequency change that caused significant inhibition of the ASR (*p* < 0.05, one-way *t-*test against zero) for control (black) and α9 KO (red) animals. Data represented as boxplots as in **(G)**. Asterisks indicate significant differences [two-way Anova, *F*_(1, 217)_ = 40.9, *p* < 0.001; Holm–Sidak *post-hoc* pairwise comparison, *t* = 6.395, *p* < 0.001]. *n* = 20 control and 13 α9 KO animals.

Previous studies demonstrated that around hearing onset, α9KO mice exhibit decreased tonotopic organization of the functional but not anatomical MNTB to LSO pathway (Clause et al., 2014). Therefore, we first tested the frequency difference limens in P14 mice (2–3 days after hearing onset). At this age, both control and α9KO mice showed a reliable acoustic startle response (ASR) in response to the startle stimulus (Figure 4). The average ASR amplitude did not differ significantly between control and α9KO mice [control: 0.68 ± 0.04 arbitrary units (AU), α9KO: 0.70 ± 0.03 AU; *p* = 0.60, Student's *t*-test; Figure 4]. The indistinguishable ASR waveforms and magnitudes in control and α9KOs indicate that startle reflex circuitry functions normally in α9KO mice.

Preceding the startle stimulus with a change in background frequency inhibited the ASR in both control and α9KO mice. As the size of the prepulse frequency change increased, the amount of inhibition also increased, reaching a plateau at around 60% inhibition. In both control and α9KO mice, the magnitude of frequency change significantly effected ASR magnitude (*p* < 0.001 in both control and α9KO, one-way ANOVA). However, the amount of inhibition elicited by small negative frequency changes (0.5–10%) was significantly less in α9KO mice while for large frequency changes (16.6 and 25%), α9KO and control mice showed similar inhibition (16.6 and 25%) (Two way ANOVA followed by Holm–Sidak *post-hoc* comparison, Figure 4). This indicates that α9KO mice showed impaired processing of small changes in frequency, although their ability to detect larger frequency steps remained intact.

The frequency difference limens were higher in α9KO mice compared to control mice. While control mice showed a significant inhibition of the ASR for frequency changes as small as 0.5%, in α9KO mice, the ASR was not significantly inhibited until the preceding frequency change reached 2% (Figure 4). Because PPI for large frequency changes was similar in α9KO and control mice, it is unlikely that alterations in the neuronal circuitry that mediates PPI are responsible for the reduced inhibition elicited by small frequency changes in α9KO mice.

#### Frequency difference limens in mature mice

**Figure 5:**
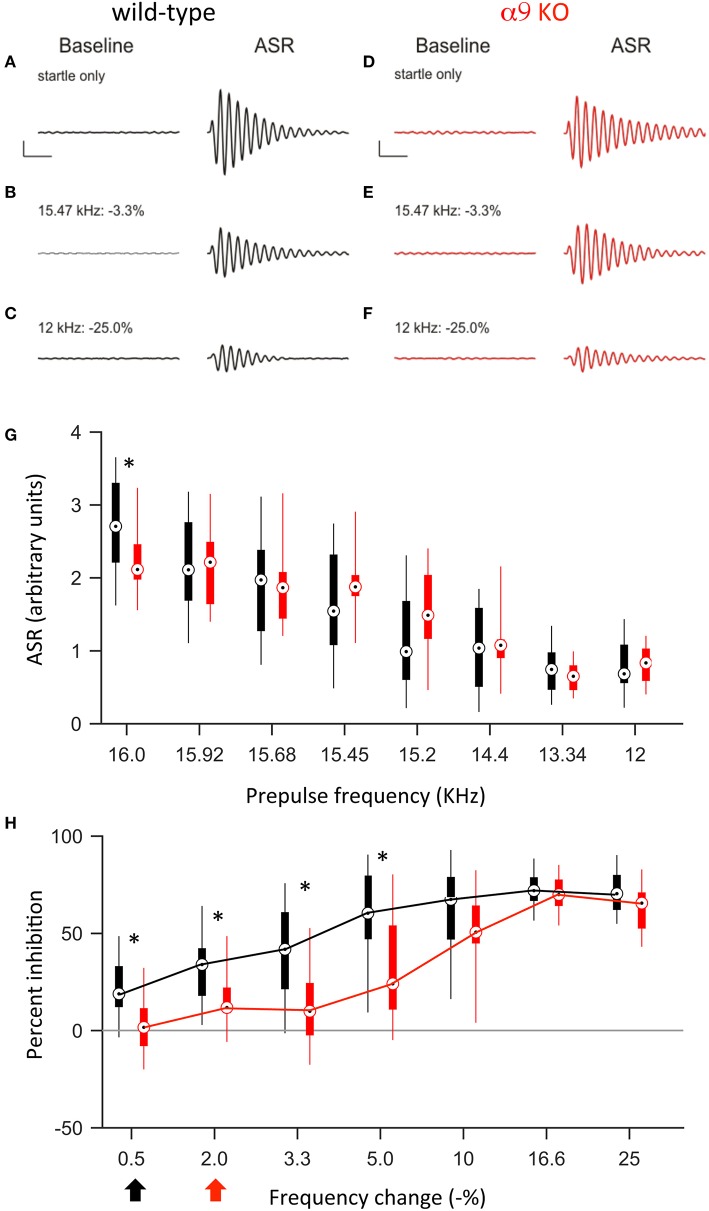
At P50, α9 KO mice continue to show impaired PPI for small, but not large, changes in frequency. **(A–F)** Examples of baseline activity and acoustic startle response for control (black; **A–C**) and α9 KO (red; **D–F**) mice. Traces represent the force measured on the platform during the first 500 ms recording period, “baseline,” and force measured during the second 500 ms recording period, “ASR” for **(A,B)** startle only trials and **(C–F)** prepulse trials with frequency steps of increasing size. Prepulse trials are labeled with the frequency of the prepulse and magnitude of the resulting frequency change. For each trial, the maximum force recorded during the second recording period, in either the positive or negative direction, was reported as the ASR. Scale bars, 1.0 arbitrary units of force, 100 ms. **(G)** ASR amplitudes for control (black) and α9 KO (red) mice for each trial type. Data are presented as boxplots with boxes representing the 25th and 75th percentiles and vertical lines indicating full data range. Two-way ANOVA indicate no significant difference tween genotypes [*F*_(1, 200)_ = 0.009, *p* = 0.93]. Asterisks indicate that ASR magnitude for α9 KO mice is significantly different than ASR magnitude for control mice for that class of trial (Holm–Sidak *post-hoc* pairwise comparison, *p* < 0.05). *n* = 15 control and 12 α9 KO animals. **(H)** Percent inhibition of the ASR caused by a prepulse frequency change of various magnitudes. Arrows indicate discrimination threshold, the smallest frequency change that caused significant inhibition of the ASR (*p* < 0.05, one-way *t-*test against zero) for control (black) and α9 KO (red) animals. Data represented as boxplots as in **(G)**. Two-way ANOVA indicates significance difference between genotypes [*F*_(1, 175)_ = 33.9, *p* < 0.001, Holm–Sidak *post-hoc* pairwise comparison, *t* = 5.820, *p* < 0.05]. *n* = 15 control and 12 α9 KO animals.

To address the question of whether the impaired frequency difference limens observed in P14 α9KO mice would persist into maturity or whether plastic mechanisms could compensate for the deficits, we tested frequency difference limens in mature animals at P50. At this age, the average ASR amplitude was larger in both control and α9KO mice, consistent with the increased weight and muscle strength of the animals (Figure 5). However, the increase in ASR amplitude was greater in control than in α9KO mice, such that the mean ASR amplitude without prepulse was significantly larger in P50 control mice than in P50 α9KO mice (control: 2.77 ± 0.16, α9KO: 2.22 ± 0.13; *p* = 0.02, Student's *t-*test). This change cannot be accounted for based on the weight of the animals because weight did not differ significantly between control and α9KO mice (P50 control: 21.5 ± 0.8 g, P50 α9KO: 21.8 ± 0.8 g; *p* = 0.80, Student's *t-*test). In addition, cochlear compound action potential (CAP) threshold sensitivity is unchanged in α9KO mice (Vetter et al., 1999; He et al., 2004), and chronic de-efferentation does not affect afferent innervation of either IHCs or OHCs (Liberman et al., 2000). Thus, it is unlikely that changes in the cochlea or in auditory nerve activity are responsible for the reduced ASR amplitudes in α9KO mice. However, because the relative degree of PPI generally remains constant despite changes in the magnitude of the ASR (Ison et al., 1997) but see (Csomor et al., 2008) and because the α9KO mice had normal PPI for large frequency changes, the reduced startle-only ASR in α9KO mice is unlikely to influence the results of difference limens testing.

Similar to P14, at P50, a change in background frequency before the startle stimulus inhibited the ASR in both control and α9KO mice, with a significant effect of prepulse magnitude on ASR amplitude (*p* < 0.001 in both control and α9KO, one-way ANOVA). However, because the ASR was reduced in α9KO mice, the magnitude of the ASR elicited by any of the prepulse frequency changes tested (15.92–12.0 kHz) did not differ between control and α9KO mice (Figure 5). Nonetheless, ASR amplitude in control mice declined more rapidly when the startle stimulus was preceded by frequency changes of increasing size. As a result, small negative changes in frequency (0.5–5.0%) elicited significantly less inhibition of the ASR in α9KO mice than in controls [Two way ANOVA, *F*_(91, 174)_ = 33.9, *p* < 0.001], consistent with the impaired frequency difference limens observed in α9KO mice at P14. Accordingly, the frequency difference limens of P50 α9KO mice were again larger than those of age-matched controls (control: 0.5%, α9KO: 2.0%).

### Intensity difference limens

**Figure 6:**
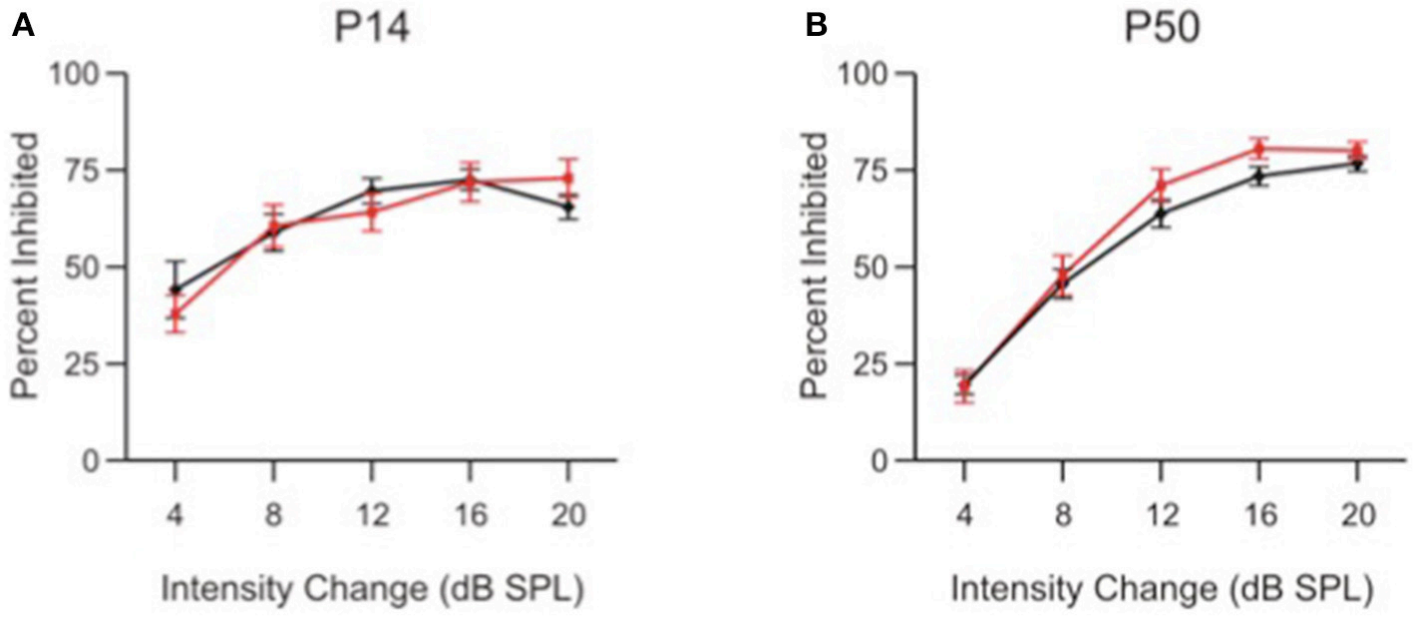
Intensity change limens is not impaired in α9 KO mice. Percent inhibition of the ASR caused by a prepulse intensity change of various magnitudes in control (black) and α9 KO (red) mice. Data represent mean ± s.e.m.; *n* = 13 control and 15 α9 KO animals at P14; *n* = 20 control and 12 α9 KO animals at P50.

The impaired frequency difference limens found in α9KO mice is consistent with a deficit in the precision of central tonotopic maps (Clause et al., 2014). Since the ability of adult α9KO mice to detect pure tones and discriminate their intensity when trained on a conditioning paradigm is normal (May et al., 2004), it is unlikely that α9KO mice show generalized deficits in other types of auditory discriminations. Therefore, to further show that the deficits in frequency difference limens of α9KO mice were not related to more generalized deficits in PPI, or to loudness cues related to the different frequencies despite our attempt to compensate for frequency-related sensation differences (Clause et al., 2011), we modified the PPI protocol to test intensity difference limens by replacing the prepulse frequency change with a prepulse intensity change (Figure 6).

At both P14 and P50, control and α9KO mice showed similar ASR inhibition at each of the intensity changes tested, indicating that responses to small intensity changes are not affected in α9KO mice, consistent with previous results from conditioning experiments (May et al., 2002). At both ages, control and α9KO mice were capable of reacting to changes in white noise intensity as small as 4 dB SPL. The specificity of PPI deficits in α9KO mice for frequency, and not intensity, along with the fact that they show equivalent levels of inhibition for large frequency changes, indicates that a generalized deficit in PPI cannot explain the impaired frequency difference limens of α9KO mice at P14 and P50.

## Discussion

In this study, we investigated whether mice lacking the α9 acetylcholine receptor subunit and thus functional MOC efferent feedback show impairments in sound localization and sound frequency difference limens. Mice tested on a conditioned lick suppression task showed variable discrimination of sound location in azimuth, and three out of four α9KO mice showed impaired MAAs. When α9KO mice were tested for frequency difference limens using PPI of the ASR, we observed poorer frequency difference limens but not intensity difference limens in mature mice as well as in mice around hearing onset.

Our results from the sound localization tests agree with previously identified auditory processing deficits and high performance variability in this mutant mouse strain (Lauer and May, 2011). Results also agree with studies from medial olivocochlear lesions that showed substantial across subject performance variability and recovery of normal function with repeated testing, suggesting that other pathways can compensate for perceptual deficits (May and McQuone, 1995; Hienz et al., 1998; May et al., 2004). The decrease in MAA in three out of four α9KO mice likely reflects the impairment in normal circuit reorganization that is present in the LSO of these mice as a result of abnormal spontaneous activity before hearing onset (Clause et al., 2014). In addition, the behavioral variability in this strain may result from dysfunction of the innate mechanism for feedback-based control of auditory nerve activity during the early period of hearing. The altered uncontrolled sound-evoked activity in α9KO mice may affect brain areas involved in sound localization beyond the LSO, whose circuit refinement is relatively insensitive to the quality of the early acoustic environment (Clause et al., 2014).

The normal MAA observed in one α9KO mouse is worth noting. Presumably, this mouse was either able to learn to use monaural sound location cues to enhance localization in the horizontal plane, or it did not experience a disruption of its binaural sound localization pathways. Future studies of MOC system effects on hearing could take individual cases into account, incorporating anatomical analysis with functional measures to further elucidate the impact of MOC system dysfunction on performance variability.

Using PPI of the ASR, we also demonstrated that α9KO mice have deficits in processing small, but not large, changes in sound frequency. These deficits were specific to sound frequency, since α9KO mice showed no deficits in detecting small sound intensity differences. The ASR amplitudes elicited by startle stimuli without a prepulse were also unchanged, consistent with the absence of α9 subunits at muscle-type nicotinic acetylcholine receptors at the neuromuscular junction, which contain α1 subunits (Kalamida et al., 2007). These normal ASR amplitudes also indicate that α9KO mice can process the broadband startle stimulus normally, which is consistent with the normal threshold sensitivity and normal CAP responses observed in these mice (Vetter et al., 1999; May et al., 2002). Finally, PPI with intensity changes and large frequency changes was normal in α9KO mice, indicating that the primary pathway mediating PPI, the inhibitory cholinergic projection from the pedunculopontine reticular nucleus to the caudal pontine reticular nucleus, also was not affected in α9KO mice. This observation is consistent with the mediation of this inhibition by muscarinic acetylcholine receptors (Koch, 1999). Therefore, the reduced ASR inhibition elicited by small frequency changes in α9KO mice is most likely due to a specific impairment in frequency processing, likely reflecting the decreased precision of central tonotopic maps in these animals (Clause et al., 2014).

The frequency difference limens deficits in α9KO mice were already present in P14 mice, only a few days after the onset of hearing (P12–P14; Song et al., 2006). This argues against the possibility that abnormal levels of sound-evoked auditory nerve activity caused by the dysfunctional MOC system caused the frequency difference limens deficits in these mice. It is more plausible that the impaired frequency difference limens in α9KO mice is caused by deficits in the refinement of functional tonotopic maps in the LSO that occurs before hearing onset. During this period, MOC fibers modulate spontaneous activity in the cochlea by inhibiting IHCs directly through activation of α9-containing nicotinic acetlycholine receptors (Glowatzki and Fuchs, 2000; Goutman et al., 2005; Roux et al., 2011). The absence of this modulation in A9KO mice results in abnormal temporal patterns of spontaneous activity and an impairment in the synaptic silencing and strengthening of inhibitory inputs to the LSO, which in turn results in less precise tonotopic maps (Clause et al., 2014). However, despite the reduced precision, maps remain tonotopically organized in α9KO mice. The specific loss of fine-scaled tonotopic precision around the time of hearing onset is consistent with the behavioral frequency difference limens data at P14; α9KO mice did not respond to small changes in frequency but showed normal responses to large frequency changes. Our result that α9KO mice at P50 still show frequency difference limens defects further indicates that the developmental hearing deficits that were acquired during the early pre-hearing period are long-lasting, perhaps resulting in life-long auditory processing impairments.

Since the efferent system may also enhance frequency tuning of the cochlea (Wiederhold, 1986; Zheng et al., 1999; Quaranta et al., 2005), one could argue that the frequency difference limens deficits in α9KO mice result from decreased frequency tuning in the cochlea. Although, earlier studies showed that acute sectioning of the efferent bundle can broaden CAP tuning curves measured with a simultaneous tone-on-tone masking paradigm (Dallos and Cheatham, 1976; Carlier and Pujol, 1982), subsequent studies showed that de-efferentation does not affect single-unit tuning curves (Bonfils et al., 1987) and that electrical stimulation of MOC fibers actually broadens auditory nerve fiber tuning curves (Guinan and Gifford, 1988). Consistent with this interpretation, complete de-efferentation in adult cats, whether acutely or chronically, has no effect on either the tuning or the sensitivity of auditory nerve fibers (Warren and Liberman, 1989; Liberman, 1990). In cats chronically de-efferented shortly after birth (P2–4), however, auditory nerve fibers show elevated thresholds. A small proportion of fibers with characteristic frequencies between ~10 and 25 kHz also show slightly reduced sharpness of tuning (Walsh et al., 1998). The authors attributed these abnormalities to a loss of normal OHC function in the chronic absence of efferent innervation, suggesting that the efferent system may play a role in the development of the OHCs' contribution to active cochlear mechanics. Functionally de-efferented α9KO mice, however, show normal cochlear sensitivity and motile OHCs (Vetter et al., 1999; He et al., 2004), suggesting that OHC function is largely undisturbed in these mice. The type of interaction between the OHCs and MOC fibers necessary for OHC maturation is unknown, although it is possible that the continued presence of efferent fibers beneath the OHCs and the release of neurotransmitter, which are maintained in α9KOs but lost in surgically de-efferented animals, plays a role.

Because the tuning of auditory nerve fibers has not been determined in α9KO mice, a potential contribution of reduced mechanical tuning to the impaired frequency difference limens of α9KO mice cannot be excluded at this time. However, reductions in the tuning of auditory nerve fibers in chronically de-efferented cats were small even for the small proportion of fibers for which they were significant. Therefore, even in the case that mechanical tuning is reduced for some frequencies in α9KO mice, the contribution of such a reduction to frequency processing deficits would likely be very small.

Behavioral and physiological deficits in hearing in noise, temporal processing, pitch discrimination, and spatial hearing have been associated with developmental language and sound processing deficits in humans (Merzenich et al., 1996; Wright et al., 1997; Gopal and Pierel, 1999; Wible et al., 2004; Banai et al., 2005). Dysfunction of the efferent auditory pathways has been implicated specific language impairment and central auditory processing disorders (Veuillet et al., 1999; Muchnik et al., 2004; Clarke et al., 2006; Sanches and Carvallo, 2006). Thus, future investigations of the role of abnormal efferent regulation of the auditory system in development and in shaping behavioral responses to sound may provide important insights into these types of disorders.

## Author contributions

AL designed, conducted, and analyzed the sound localization experiments. AC designed, conducted, and analyzed the frequency and intensity limens experiments. KK conceived and designed frequency and intensity limens experiments. All authors discussed the results and contributed to writing the manuscript.

### Conflict of interest statement

The authors declare that the research was conducted in the absence of any commercial or financial relationships that could be construed as a potential conflict of interest.
